# 

**Published:** 1860-01

**Authors:** 


New Series, Vol. 3, No. 1. Whole Series, Vol. 17, No. 1.

### THE CHICAGO MEDICAL JOURNAL.

DANIEL BRAINARD, M. D.,

EPHRAIM INGALS, M. D.,

EDITORS AND PROPRIETORS.

W. GODFREY DYAS, M. D., Assistant Editor.

JANUARY, 1860.

CHICAGO:
HYATT BROTHERS, BOOK AND JOB PRINTERS, 23 LAKE STREET,
To whom all advertisements for insertion in the Journal may be addressed.

PUBLISHED MONTHLY, AT TWO DOLLARS PER ANNUM, IN ADVANCE.

